# Complex Changes in von Willebrand Factor-Associated Parameters Are Acquired during Uncomplicated Pregnancy

**DOI:** 10.1371/journal.pone.0112935

**Published:** 2014-11-19

**Authors:** Danielle N. Drury-Stewart, Kerry W. Lannert, Dominic W. Chung, Gayle T. Teramura, James C. Zimring, Barbara A. Konkle, Hilary S. Gammill, Jill M. Johnsen

**Affiliations:** 1 Research Institute, Puget Sound Blood Center, Seattle, Washington, United States of America; 2 Department of Laboratory Medicine, University of Washington, Seattle, Washington, United States of America; 3 Department of Medicine, University of Washington, Seattle, Washington, United States of America; 4 Department of Obstetrics and Gynecology, University of Washington, Seattle, Washington, United States of America; 5 Division of Clinical Research, Fred Hutchinson Cancer Research Center, Seattle, Washington, United States of America; National Cerebral and Cardiovascular Center, Japan

## Abstract

**Background:**

The coagulation protein von Willebrand Factor (VWF) is known to be elevated in pregnancy. However, the timing and nature of changes in VWF and associated parameters throughout pregnancy are not well understood.

**Objectives:**

To better understand the changes in VWF provoked by pregnancy, we studied VWF-associated parameters in samples collected over the course of healthy pregnancies.

**Methods:**

We measured VWF antigen (VWF:Ag), VWF propeptide (VWFpp), Factor VIII (FVIII), and ADAMTS13 activity in samples collected from 46 women during pregnancy and at non-pregnant baseline. We also characterized pregnant vs. non-pregnant VWF multimer structure in 21 pregnancies, and performed isoelectric focusing (IEF) of VWF in two pregnancies which had samples from multiple trimesters.

**Results:**

VWF:Ag and FVIII levels were significantly increased during pregnancy. ADAMTS13 activity was unchanged. VWFpp levels increased much later in pregnancy than VWF:Ag, resulting in a progressive decrease in VWFpp:Ag ratios. FVIII:VWF ratios also decreased in pregnancy. Most pregnancies exhibited a clear loss of larger VWF multimers and altered VWF triplet structure. Further evidence of acquired VWF qualitative changes in pregnancy was found in progressive, reversible shifts in VWF IEF patterns over gestation.

**Conclusions:**

These data support a new view of pregnancy in which VWF can acquire qualitative changes associated with advancing gestational age. Modeling supports a scenario in which both increased VWF production and doubling of the VWF half-life would account for the data observed. We propose that gestation induces a prolongation in VWF survival, which likely contributes to increased total VWF levels and altered VWF structure.

## Introduction

von Willebrand Factor (VWF) is an abundant plasma glycoprotein which stabilizes Factor VIII (FVIII, the protein deficient in Hemophilia A) in circulation and adheres at sites of vascular injury, where it recruits platelets. VWF levels are widely variable in the healthy population, with a “normal” range spanning 40–200 U/dL [1]. Variation in VWF levels can be clinically significant. High levels of VWF are associated with thrombosis [2], while low VWF levels are associated with an increased risk of bleeding and the diagnosis of von Willebrand Disease (VWD) [3].

VWF is exclusively synthesized in endothelial cells and megakaryocytes as a prepro-VWF molecule which undergoes extensive post-translational modification, including cleavage of the signal peptide, dimerization, glycosylation, cleavage of the propeptide (VWFpp), and multimerization (reviewed in Johnsen and Ginsburg [3]). VWFpp and mature VWF (VWF:Ag) are secreted at equimolar ratios, but after secretion each circulates independently with distinct half-lives [4], [5]. Newly secreted VWF:Ag is a very large, hemostatically active multimer which is cleaved in circulation into smaller multimers by the plasma protease a disintegrin and metalloproteinase with thrombospondin motifs-13 (ADAMTS13) [6]. Deficiency in ADAMTS13 leads to an overabundance of ultra-large VWF multimers and is associated with thrombotic thrombocytopenic purpura [7].

Pregnancy is a hypercoagulable state [8]–[13] in which elevations in pro-coagulant proteins have been observed [14], including marked increases in VWF and FVIII [14]–[16]. Interestingly, one cross-sectional study observed that VWFpp levels were elevated in pregnancy, but did not rise to the same degree as VWF:Ag, resulting in VWFpp:Ag ratios as low as 0.5 in late gestation [15]. However, the wide variation in VWF parameters in normal populations has confounded determinations of the size and timing of changes in VWF:Ag, FVIII, and VWFpp in pregnancy.

We sought to characterize the timing of changes in VWF and VWF-associated parameters longitudinally over the course of pregnancy. We measured VWF:Ag, FVIII:Ag (and FVIII:C), VWFpp, and ADAMTS13 activity and analyzed these data relative to each other and to gestational age. We further hypothesized that pregnancy provokes not only a quantitative increase in VWF, but also acquired qualitative changes which could be reflected in the VWFpp:Ag ratio, VWF:FVIII ratio, VWF multimer structure, and VWF isoelectric focal point.

## Materials and Methods

### Human Samples

De-identified samples of plasma and whole blood or DNA were obtained from a healthy pregnancy repository for which samples were collected longitudinally during pregnancy by Drs. Nelson and Gammill (Fred Hutchinson Cancer Research Center and University of Washington). This repository prospectively recruits healthy women with a singleton pregnancy seeking obstetric care in Seattle, Washington, USA. To ensure restriction of the repository to women with a normal obstetric outcome, medical records and a self-reported questionnaire were reviewed after delivery for obstetric clinical information. Subjects with pregnancy complications, including preterm birth (spontaneous or indicated), hypertensive disorders of pregnancy (e.g. preeclampsia, gestational hypertension, or other complications (fetal growth restriction, diabetes, placenta previa, or placental abruption)), were excluded from the repository. No subjects experienced symptomatic bleeding or thrombosis during pregnancy or in the postpartum period. The samples studied were acquired between November 1995 and January 2011. Samples were drawn in heparin or citrate at a routine clinic/study visit, processed for whole blood and platelet-poor plasma, and aliquots were snap frozen and stored at −80C. All plasma samples received from the repository had not been previously thawed. To be included in this study, repository pregnancies had to have plasma samples drawn at a baseline and third trimester time point. Non-pregnant baseline samples were drawn within one year of gestation, either pre-pregnancy or≥6 weeks postpartum [17]; for subjects with pre-pregnancy and postpartum samples the average number of days between the first and last blood draws was 477+/−81 days (maximum 621 days). When available, plasma samples from the first and/or second trimesters were also included, as well as samples from 38 weeks gestation if the subject could be verified not to have been in labor. Individual data measurements for each subject at each time point are reported in Table S1. This work studied human repository samples; subjects provided written informed consent to participate in studies, and samples were maintained in the repository with the oversight and approval of the Fred Hutchinson Cancer Research Center Institutional Review Board (FHCRC IRB). This work in the repository was approved by the FHCRC IRB.

### VWF Antigen (VWF:Ag), VWF propeptide (VWFpp), and Factor VIII (FVIII) Measurements

VWF:Ag and VWFpp were determined by enzyme-linked immunosorbent assays (ELISAs) per the manufacturer's instructions (GTI Diagnostics). Clinical assays of VWF function were not performed due to sample volume constraints. FVIII antigen (FVIII:Ag) levels were determined by ELISA (Affinity Biologicals) in all samples. The anticoagulant used during sample acquisition (heparin or citrate) was controlled for in each of these assays. A FVIII activity assay (FVIII:C) was performed for all citrated samples on unthawed aliquots using a one-stage assay optimized for the anticipated high range of FVIII:C activity. We found no difference between FVIII:Ag and FVIII:C measurements either at baseline or in the third trimester (p = 0.928, 0.929), which addressed any concern of artifactual suppression of FVIII activity by high VWF levels [18]. As FVIII:Ag measurements were available for all subjects, FVIII:Ag measurements were chosen to represent the FVIII values and are reported as “FVIII” hereafter.

### ADAMTS13 Activity

Plasma ADAMTS13 activity was measured via cleavage of an enzyme-linked peptide substrate as previously described [19]. Standards were generated in citrate and heparin as appropriate for each sample's anticoagulant.

### VWF Multimer Analysis

Plasma samples were normalized to 50 U/dL VWF in VWF-deficient plasma (Affinity Biologicals) based upon VWF:Ag ELISA results. VWF multimer gels (1% and 2.6% (w/v) SeaKem HGT(P) agarose (Lonza)) were loaded with normalized plasmas diluted in water and 2x sample buffer (50 mM tris base, 6 M urea, 10 mM EDTA, 2% SDS, 0.03% bromophenol blue, pH 6.8) to a final dilution of 1∶20 and incubated for 20 minutes at room temperature. Gels were run for 20 hours at room temperature in running buffer (50 mM tris base, 384 mM glycine, 0.1% SDS) and blotted to a PVDF membrane. Membranes were blocked in 5% milk/1x TBS shaking and probed with HRP-conjugated rabbit polyclonal anti-VWF (Dako) at 1∶1000 and developed using chemiluminescent substrate (Millipore). Digital images were acquired and densitometry waveforms generated using ImageQuant 350 (GE). Assessments of multimer structure were determined by two independent observers using both the original gel images and overlays of densitometry tracings; a change in multimer structure was only assigned if independently determined by both observers.

### Isoelectric Focusing (IEF)

Plasma samples were normalized using VWF-deficient plasma to the lowest sample concentration (64 U/dL); pooled normal human plasma and plasma-derived VWF/FVIII concentrate (Humate-P: CSL Behring) were used as positive controls, while VWF-deficient plasma (Affinity Biologicals) was used as a negative control. After normalization, samples were diluted 1∶20 in water and heated at 60°C for 20 minutes. After a further 1∶1 dilution with loading buffer (Invitrogen, pH 3–7), a total of 0.25 µl of plasma was added to each well. Isoelectric focusing (IEF) of native plasma was performed in precast 5% acrylamide gels pH range 3–7 per the manufacturer's instructions (Invitrogen), blotted to PVDF, detected by immunoblotting (HRP-conjugated rabbit anti-human VWF, Dako), and analyzed as described for the VWF multimer analysis.

### ABO Blood Group Determination

DNA was extracted from frozen whole blood aliquots using the DNeasy kit (Qiagen) per the manufacturer's instructions. SNP-specific PCR for ABO designation was performed using the Red Cell EZ Type (GTI Diagnostics). A1, A2, B, O1, and O2 genotypes were assigned per the manufacturer's instructions and correlated with clinical ABO blood types captured in the repository data, when available.

### Calculations and Statistics

Differences between third trimester and baseline samples were calculated by the Student's two-tailed paired *t*-test or by the Wilcoxon Signed Rank Test, as appropriate depending upon data distribution characteristics. Differences across all time points were determined by ANOVA followed by a Tukey post-test or the Kruskal-Wallis ANOVA on Ranks followed by the Dunn post-test, as indicated. All statistical analyses were carried out using SigmaPlot 12.0 (Systat Software, Inc.).

The VWFpp:Ag Ratio was calculated by dividing VWFpp by VWF:Ag values. Similarly, the FVIII:VWF ratio was calculated by dividing FVIII by VWF:Ag. VWF half-life was modeled in third trimester and postpartum samples using the VWFpp as an indicator of VWF production. [20], [21] To calculate t_1/2VWF:Ag_ in the third trimester, we assumed single-order, single compartment kinetics and conservatively biased our calculations against finding a prolongation in VWF survival by setting the baseline t_1/2VWF:Ag_ at the high end of normal (12 hours) [22]–[24] and t_1/2VWFpp_ (assumed to be stable) at the low end of normal (2 hours). [4] To account for the plasma volume expansion during pregnancy, we set V_Dbaseline_ = 260dL and V_D3rdTrimester_ = 315–385dL. [25] We then used the equation: Protein_kin_ = V_D_ x K_e_ x C_ss_, where K_e_ = ln(2)/t_1/2_.

## Results

Forty-six healthy pregnancies (patient characteristics are described in Table 1) qualified for the study by having non-pregnant baseline and third trimester samples available for analysis. An analysis of the measured parameters relative to the date of sample acquisition revealed no association, supporting maintenance of the integrity of the samples during storage. Preconception and>6 weeks postpartum samples were available for fourteen pregnancies. An analysis comparing VWF:Ag, VWFpp, and FVIII in preconception and postpartum samples from the same pregnancies found no differences (Table 2).

**Table 1:** Patient characteristics (n = 46 women, 179 samples).

	Mean ± SD or %
**Characteristics**	
Maternal Age at Delivery	32.7±3.8 years
Gestational Age at Delivery	39.8±1.3 weeks
Gravidity	1.7±0.8
Parity	0.5±0.6
**Race**	
Caucasian	82.6%
Asian/Pacific Islander	8.7%
Hispanic	2.2%
Other	6.5%
Cesarean Delivery (%)	28.2%
Birthweight (grams)	3475±441
Fetal sex (% male)	43%
**Timing of Sample Collection (GA in weeks)**	
1^st^ trimester samples	9.3±3.4
2^nd^ trimester samples	21.9±2.3
3^rd^ trimester samples	35.2±2.7

**Table 2:** VWF-associated parameters in 46 women during pregnancy and at non-pregnant baseline (Fig 1).

	Time Point	Mean ± SD	Median	25^th^ Percentile	75^th^ Percentile
**VWF:Ag (U/dL)**	Baseline	80.7+/−26.6	80.6	59.4	94.8
	1^st^ trimester**^†^**	120.3+/−41.9	114.5	92.8	150.6
	2^nd^ trimester**^†^**	139.9+/−47.3	131.64	98.7	174.4
	3^rd^ trimester**^†,1^**	191.3+/−70.3	192.2	134.0	245.6
**VWFpp (U/dL)**	Baseline	130.1+/−31.5	127.1	104.4	152.7
	1^st^ trimester	143.4+/−38.7	135.7	116.9	169.4
	2^nd^ trimester	141.2+/−32.2	138.7	116.0	154.2
	3^rd^ trimester**^†^**	157.7+/−43.1	164.2	120.8	188.0
**FVIII:Ag (U/dL)**	Baseline	117.5+/−33.1	111.1	90.3	141.4
	1^st^ trimester	133.4+/−40.0	128.4	107.6	160.0
	2^nd^ trimester**^†^**	169.6+/−53.1	154.8	125.0	203.8
	3^rd^ trimester**^†,1^**	199.2+/−56.1	196.2	156.7	232.5
**VWFpp:Ag Ratio**	Baseline	1.73+/−0.54	1.57	1.27	2.16
	1^st^ trimester**^†^**	1.25+/−0.26	1.21	1.04	1.43
	2^nd^ trimester**^†^**	1.07+/−0.24	1.07	0.88	1.27
	3^rd^ trimester**^†,1^**	0.89+/−0.26	0.84	0.69	1.00
**FVIII:VWF Ratio**	Baseline	1.54+/−0.48	1.43	1.19	1.72
	1^st^ trimester**^†^**	1.17+/−0.31	1.14	0.95	1.33
	2^nd^ trimester	1.25+/−0.25	1.26	1.06	1.43
	3^rd^ trimester**^†^**	1.13+/−0.35	1.09	0.87	1.30

Trimesters (n): 1st trimester (24), 2nd trimester (38), 3rd trimester (46), Non-pregnant baseline (46).

Statistical analysis: One-way ANOVA followed by all pairwise multiple comparisons: ^†^p<0.05 Difference from Baseline; 1 p<0.05 Difference from 1st trimester.

### VWF:Ag, FVIII, and VWFpp

**Figure 1:**
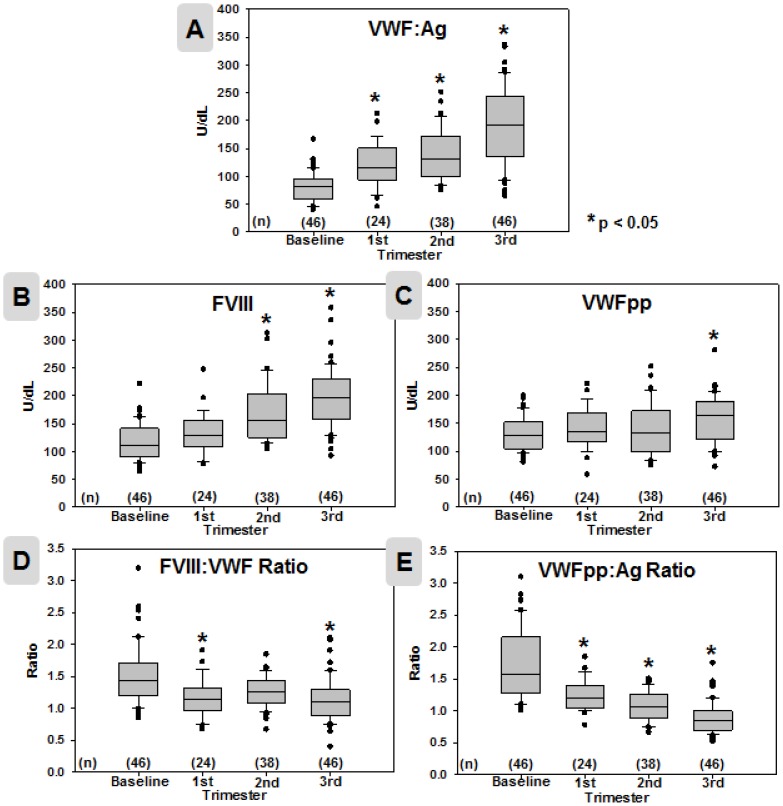
VWF-associated parameter data in 46 pregnancies. Values for VWF:Ag (**A**), FVIII (**B**), VWFpp (**C**), FVIII:VWF Ratio (**D**), VWFpp:Ag Ratio (**E**) are presented at each time point (non-pregnant baseline n = 46, 1^st^ trimester n = 24, 2^nd^ trimester n = 38, 3^rd^ trimester n = 46). The median, 25^th^ and 75^th^ percentiles (boxed), 1 SD (bars), and individual outlying data points (black dots) are shown for each parameter at each time point. Statistically significant differences from baseline (*p*<0.05 by one-way ANOVA followed by all pairwise multiple comparisons) are indicated by asterisks (*). Values can be found in Table 2.

As expected, both VWF:Ag and FVIII levels significantly increased during pregnancy to values more than double baseline in the third trimester (Table 3). VWFpp levels trended slightly higher during pregnancy; a paired comparison between baseline and 3^rd^ trimester values revealed a significant difference (Table 3). However, when all data were analyzed longitudinally, VWFpp levels were not statistically significantly higher than baseline until the third trimester (Fig 1, Tables 2 and 4).

**Table 3:** Hemostatic parameters (± SD) at baseline and in the 3^rd^ trimester of 46 healthy pregnancies.

	Baseline	3^rd^ Trimester	*p* value†
** VWF:Ag (U/dL)**	80.7+/−26.6	191.2+/−70.3	<0.001
** VWF:pp (U/dL)**	130.1+/−31.4	157.7+/−43.0	<0.001
** FVIII:Ag (U/dL)**	117.5+/−33.1	199.2+/−56.1	<0.001
**ADAMTS13 (% Activity)**	121.7+/−34.4	117.7+/−28.7	0.33
** VWFpp:Ag Ratio**	1.73+/−0.54	0.89+/−0.26	<0.001
** FVIII:VWF Ratio**	1.54+/−0.48	1.13+/−0.35	<0.001

† - Wilcoxon Signed Rank Test.

**Table 4:** Changes in VWF-associated parameters (relative to non-pregnant baseline) in 46 pregnancies (Fig 2).

	Time Point	Mean (%) ±1 SD	Median	25^th^ Percentile	75^th^ Percentile
**VWF:Ag**	Pre-pregnancy	109±27	100	96	130
	1^st^ trimester^†^	154±61	136	126	160
	2^nd^ trimester^†,Pre^	188±70	181	146	219
	3^rd^ trimester^†,Pre,1^	246±94	228	188	299
	38 weeks^†,Pre,1^	331±189	294	212	374
	Postpartum	100	100	—	—
**VWFpp**	Pre-pregnancy	97±16	100	84	102
	1^st^ trimester	109±34	104	81	120
	2^nd^ trimester	114±24	117	101	134
	3^rd^ trimester	126±44	116	93	150
	38 weeks^†,Pre^	155±79	134	124	183
	Postpartum	100	100	—	—
**FVIII:Ag**	Pre-pregnancy	98±10	100	93	106
	1^st^ trimester	122±44	113	100	130
	2^nd^ trimester^†,Pre^	149±36	148	125	166
	3^rd^ trimester^†,Pre,1^	177±56	168	148	193
	38 weeks^†,Pre,1^	175±55	169	134	210
	Postpartum	100	100	—	—
**VWFpp:Ag Ratio**	Pre-pregnancy	93±26	98	76	100
	1^st^ trimester^†^	76±25	74	57	93
	2^nd^ trimester^†,Pre^	65±18	64	53	78
	3^rd^ trimester^†,Pre^	54±14	53	43	64
	38 weeks^†,Pre^	53±27	47	40	55
	Postpartum	100	100	—	—
**FVIII:VWF Ratio**	Pre-pregnancy	96+/−28	100	70	100
	1^st^ trimester^†^	79+/−41	76	65	90
	2^nd^ trimester^†^	85+/−24	87	63	98
	3^rd^ trimester^†^	77+/−23	75	61	86
	38 weeks^†,Pre,2^	61+/−17	60	47	71
	Postpartum	100	100	—	—

Sample numbers (n): Pre-pregnancy (14), 1^st^ trimester (24), 2^nd^ trimester (38), 3^rd^ trimester (46), Postpartum (41).

Statistical analysis: One-way ANOVA followed by all pairwise multiple comparisons: **^†^** *P*<0.05 Difference from Postpartum; ^Pre^ *P*<0.05 Difference from Pre-pregnancy; ^1^ *P*<0.05 Difference from 1^st^ trimester; ^2^ *P*<0.05 Difference from 2^nd^ trimester; ^3^ *P*<0.05 Difference from 3^rd^ trimester.

**Figure 2:**
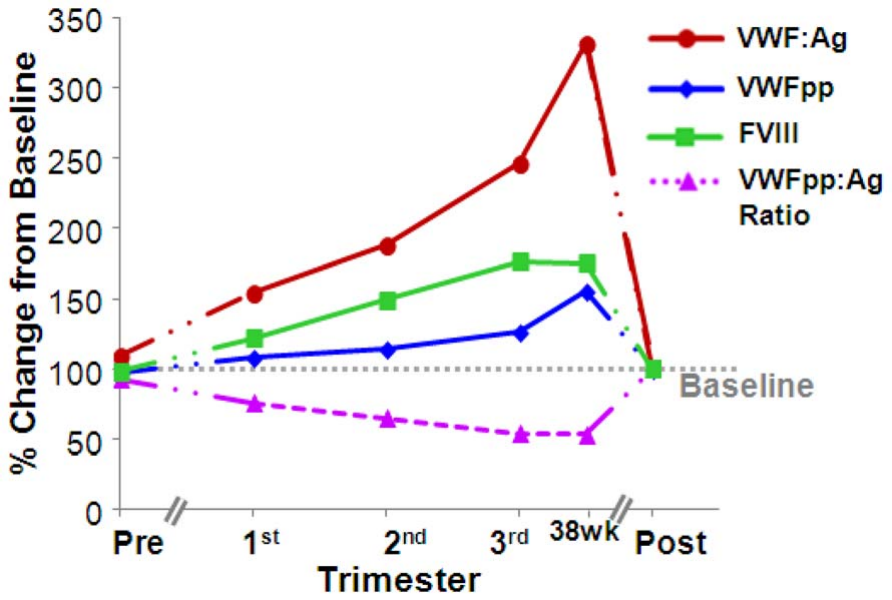
Changes in VWF-associated parameters from baseline in 46 pregnancies. The changes in VWF parameters normalized to baseline are displayed longitudinally over pregnancy for time points sampled in pre-pregnancy n = 14, 1^st^ trimester n = 24, 2^nd^ trimester n = 38, 3^rd^ trimester n = 44, 38wks n = 12, and postpartum n = 41. Asterisks (*) indicate *p*<0.05 compared to postpartum values (one-way ANOVA, all pairwise multiple comparisons). Standard deviations, means, 25^th^, and 75^th^ percentiles are omitted for clarity and can be found in Table 4.

The increase in VWF:Ag was disproportionate to VWFpp (Figs 1 and 2), with a significant decrease in the VWFpp:Ag ratio to nearly half that of the baseline ratio by the third trimester (Table 3). Analysis of the changes in VWF:Ag and VWFpp levels longitudinally over pregnancy revealed temporal distinctions in the shifts of these two parameters (Figure 2). Notably, VWF:Ag was significantly elevated in the 1^st^ trimester, while VWFpp was not significantly elevated from baseline until late in the 3^rd^ trimester. This resulted in a trend of progressive decreases in the VWFpp:Ag ratio beginning early in pregnancy, becoming statistically significant by the 2nd trimester and markedly decreasing by the third trimester. Mean, median, and quartile values are shown in Tables 2 and 4.

### Modeling of VWF production and survival

To investigate if VWF production alone could account for the increase in VWF:Ag observed in pregnancy, we calculated VWF production relative to measured VWF:Ag and predicted VWF half-life, taking into account the volume expansion of pregnancy. As shown in Table 5, the calculated VWF propeptide production (VWFpp_kin_) increased in the third trimester, consistent with a marked increase in VWF production. However, the average measured VWF:Ag in the third trimester (191 U/dL) was well in excess of the VWF value predicted by production alone (101 U/dL). Taking into account the calculated increase in VWF production in the third trimester and the dilution effects of volume expansion during pregnancy, an extension of the t_1/2VWF:Ag_ from 12 to 24 hours would account for the excess VWF:Ag observed.

**Table 5:** Calculated VWF excess and VWF half-life in pregnancy.

	Baseline [5]	3rd Trimester	Fold Increase
***Expected vs. Observed VWF***			
Calculated VWF production (U/hr)	1202	2194	1.8
Observed VWF concentration (U/dL)	80	191	2.4
Expected VWF concentration (U/dL)		101	1.3
***VWF_t1/2_ which would account for observed VWF excess***			
VWF:Ag_t1/2_ (hours)	12	24	2.0

### FVIII:VWF ratio

Although FVIII levels significantly increased across gestation, we observed the increases in FVIII to lag behind VWF:Ag, with the decrease in the FVIII:VWF ratio becoming most pronounced in the third trimester (Figs 1 and 2, Tables 2 and 4).

### ABO blood group

The distribution of ABO blood types (Table 6) was 28% O (n = 13), 37% A (n = 17), 28% B (n = 13), and 6.5% AB (n = 3). In a subgroup analysis by ABO blood type (types O, A, and B) comparing baseline and 3^rd^ trimester measures, most of the changes observed during pregnancy remained statistically significant. In blood type O subjects, VWFpp trended upward, but the change was not statistically significantly different from baseline (Table 7). Additionally, the change in the FVIII:VWF ratio was more modest in blood type O subjects, but still statistically significant (p = 0.04). These data suggest that ABO blood type does not play a major role relative to VWF changes in pregnancy. However, this study is underpowered to detect modest associations between ABO and changes in VWF parameters provoked by pregnancy.

**Table 6:** ABO types of the 46 pregnant subjects.

Genotype	# of Subjects	ABO Type	% of Subjects	% of US Population [53]
O_1_O_1_	13	O (n = 13)	28.3%	48%
O_1_A_1_	12	A (n = 17)	37.0%	37%
O_1_A_2_	3			
A_1_A_1_	1			
A_1_A_2_	1			
O_1_B	12	B (n = 13)	28.3%	11%
O_2_B	1			
A_1_B	2	AB (n = 3)	6.5%	4%
A_2_B	1			

**Table 7:** VWF parameter changes in pregnancy from non-pregnant baseline by ABO blood group.

Parameter	ABO	Baseline	3^rd^ Trimester
**VWF:Ag**	O**^†^**	69±18	169±80
	A**^†^**	76±21	182±51
	B**^†^**	86±24	199±68
	AB*	137±27	302±30
	non-O**^†^**	85±28	200±65
**VWFpp**	O	117±29	136±42
	A**^†^**	130±31	158±40
	B**^†^**	134±29	164±35
	AB*	168±31	218±55
	non-O**^†^**	135±31	166±41
**FVIII**	O**^†,#^**	96±26	182±62
	A**^†^**	113±26	198±49
	B**^†^**	136±35	206±59
	AB*	156±19	249±44
	non-O**^†^**	126±32	206±53
**VWFpp:Ag**	O**^†^**	1.79±0.57	0.89±0.24
	A**^†^**	1.82±0.58	0.91±0.26
	B**^†^**	1.66±0.47	0.90±0.29
	AB*	1.22±0.04	0.71±0.11
	non-O**^†^**	1.70±0.53	0.89±0.26
**FVIII:VWF**	O**^†,#^**	1.46+/−0.38	1.23+/−0.49
	A**^†^**	1.54+/−0.36	1.12+/−0.22
	B^†,#^	1.73+/−0.69	1.12+/−0.37
	AB*	1.15+/−0.10	0.82+/−0.07
	non-O**^†,#^**	1.58+/−0.52	1.09+/−0.29

Sample numbers (n): O (13); A (17); B (13); AB (3); non-0 (33).

Statistical analysis: **^†^** *P*<0.05 difference in 3^rd^ trimester from baseline; *AB blood group data was omitted from the statistical analysis due to small sample size; Analysis were performed by either the Student's two-tailed paired t-test or, as indicated ^#^, the Wilcoxon Signed Rank Test.

### VWF multimer structure

**Figure 3:**
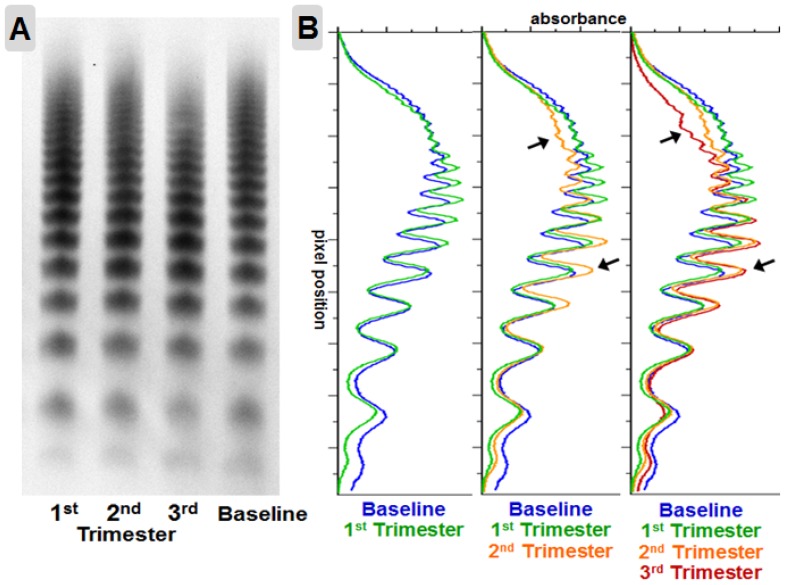
Loss of higher weight VWF multimers in pregnancy. A normalized 1% multimer gel (**A**) and densitometry (**B**) of plasma VWF multimers from a single pregnancy demonstrating the most commonly observed pattern of shifted VWF multimers (arrows) in the second trimester (orange) and third trimester (red) compared to baseline VWF (blue).

**Figure 4:**
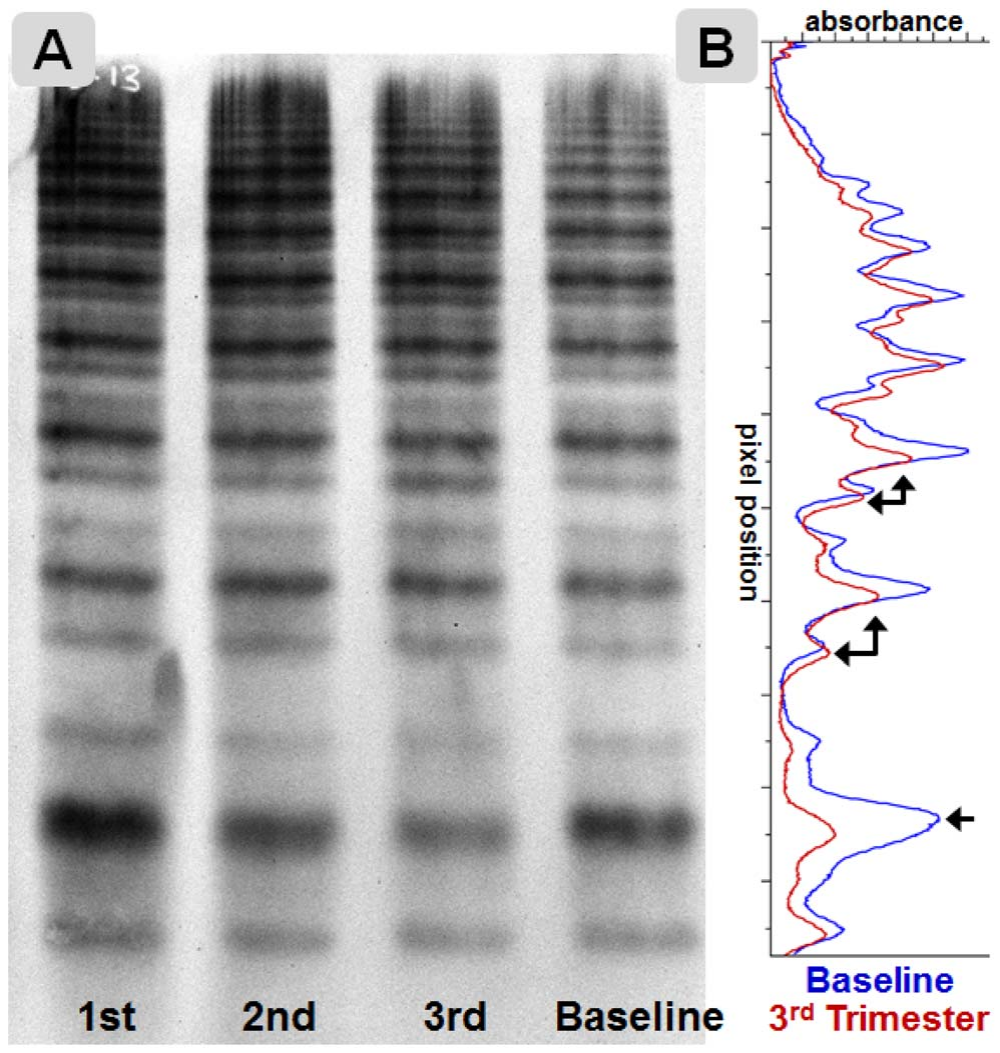
Change in VWF triplet structure in pregnancy. A normalized high resolution 2.6% multimer gel (**A**) and densitometry (**B**) of the same pregnancy shown in Fig 3 demonstrating the most commonly observed pattern of shifted VWF triplet structure with relative loss of height of the major band in the 3^rd^ trimester (red) compared to baseline (blue).

VWF multimer analysis on standard (1%) and high (2.6%) resolution gels was performed in 21 randomly selected pregnancies comparing third trimester to baseline samples. A pattern of relative loss of high molecular weight VWF multimers and gain of intermediate VWF multimers (Fig 3) was observed in 81% (17/21) of pregnancies analyzed. Additionally, 81% of subjects (n = 17/21) exhibited decreased prominence of the central triplet band with redistribution to the satellite bands in the lower order triplets (Fig 4). Interestingly, there was heterogeneity in the multimer patterns observed, and 5 subjects (28%) exhibited a marked change in only one multimer pattern (usually with changes in the other multimer pattern scored by one but not both observers), while 3 subjects (14%) scored no clear change in either higher order or triplet VWF multimer structure by both observers. These results indicate that acquired multimeric changes in VWF are common during pregnancy.

### ADAMTS13 activity

ADAMTS13 activity was unchanged in third trimester samples compared to baseline (Table 3), indicating that changes observed in VWF levels and multimer structure are unlikely to be due to acquired changes in ADAMTS13 activity.

### VWF isoelectric focusing point(s)

**Figure 5:**
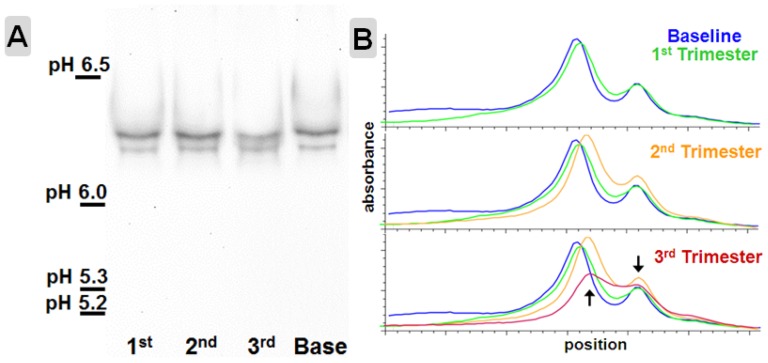
Shift in isoelectric focus (IEF) pattern in pregnancy. **A**. A normalized plasma isoelectric focusing gel from the same pregnancy as shown in Figs 3 and 4 immunoblotted for VWF. **B**. Densitometry tracings demonstrate the progressive shift in VWF isolectric focus in the 2^nd^ (orange) and 3^rd^ (red) trimesters. In the 3^rd^ trimester, the band volume ratio of VWF in the doublet has shifted (3∶1 to nearly 2∶1) and the upper VWF band has shifted towards the lower pH focused VWF band.

VWF isoelectric focusing was performed in two pregnancies which had plasma samples available for multiple trimesters. As shown in one of the pregnancies in Fig 5, at non-pregnant time points the isoelectric point of VWF appears as a distinct doublet between pH 6.0 and 6.5 with an upper:lower band volume ratio of approximately 3∶1. In pregnancy, the VWF isoelectric focusing point of the upper band progressively shifted markedly towards the lower band by the third trimester (Fig 5). These data indicate that VWF acquires reversible molecular changes during pregnancy.

## Discussion

Pregnancy is a procoagulant state thought to reflect the result of adaptive evolution in the face of considerable bleeding risks during delivery and in the immediate postpartum period. The clinical significance of the hemostatic challenge posed by pregnancy is illustrated by the fact that peripartum hemorrhage is the leading cause of maternal mortality in the developing world [26] and remains a significant challenge even in the setting of advanced medical care [27], [28]. However, this presumably adaptive procoagulant state of pregnancy appears to pose its own dangers, as pregnancy is also associated with a significantly increased risk of thromboembolic disease [8], [9], [12], [13] and pregnancy-associated vascular complications impact millions of women each year [11], [29]. In the non-pregnant state, high and low levels of VWF and FVIII are well established to be associated with thrombotic and bleeding risk, respectively [30]–[34] making VWF and FVIII excellent candidates to both mitigate maternal hemorrhage and participate in the pathogenesis of the vascular disorders of pregnancy. To our knowledge, VWF, FVIII, and the VWF-associated parameters VWFpp and ADAMTS13 activity have not been studied longitudinally in pregnancy, and investigations of VWF in pregnancy [31] have focused on measuring the levels of VWF antigen. Here, we report a more comprehensive and longitudinal characterization of the changes in VWF-associated coagulation parameters acquired during pregnancy, and further offer evidence that acquired, reversible molecular changes in VWF can be provoked by pregnancy.

In this study, we found VWF:Ag levels markedly increased with advancing gestational age, as expected from previous cross-sectional studies [15]. Although the concentration of VWFpp was not statistically significantly increased until very late in pregnancy, we calculate that the total VWFpp is increased over pregnancy when taking into account the volume expansion which occurs during pregnancy.

Interestingly, the VWFpp did not increase proportionate with VWF:Ag, resulting in a marked decline in the VWFpp:Ag ratio to nearly half of baseline by the third trimester. This striking shift in the VWFpp:Ag ratio is consistent with the findings of Sie et al. [15], although our interpretation of the data differ. Sie et al. concluded that the decrease in the VWFpp:Ag ratio could be attributed to increased VWF production in the face of the differences in half-life of VWFpp (2–3 hours) and VWF:Ag (8–12 hours) [35]. A change in VWFpp:Ag ratio would indeed be predicted to occur when changes in VWF homeostasis occur over hours to days, and this scenario as has been proposed to explain in the shift in the VWFpp:Ag ratio observed during acute illnesses, such as malaria [36] and sepsis [37]. However, during pregnancy the changes in VWF are sustained over a prolonged period relative to the half-lives of the VWFpp and VWF:Ag proteins, resulting in both proteins circulating essentially at steady state. Taking both the timing of change and the volume expansion of pregnancy into account, we interpret the steady VWFpp levels to be consistent with increased VWF production and the shift in the VWFpp:Ag ratio to reflect a prolongation in VWF survival, similar to the inverse correlation between VWFpp:Ag ratio and VWF half-life observed in non-pregnant states [38]–[40]. Interestingly, we found the decrease in the VWFpp:Ag ratio begins early in pregnancy, becomes statistically significant in the second trimester, and drops even further in the third trimester, supporting progressive changes which influence the VWFpp:Ag ratio as gestation advances.

Over the course of these pregnancies, FVIII levels also markedly increased, as expected. However, elevations in FVIII lagged modestly relative to VWF, resulting in a significant decrease in the FVIII:VWF ratio, particularly when data was analyzed relative to the subject's own baseline (Fig 2). This data supports a shift in FVIII:VWF stoichiometry, with fewer FVIII molecules relative to VWF. Interestingly, this may not be due to an inability to achieve a high FVIII level, as average FVIII levels>200 U/dL are reported in settings of acute events such as myocardial infarction, where FVIII can increase proportionately with, or even greater than, VWF [41]. A review of the literature identified several studies which corroborate our observation of decreased FVIII:VWF ratios in pregnancy [42]. Furthermore, the FVIII:VWF ratio was also reported to be more dramatically decreased in pregnancies complicated by preeclampsia [43]–[46], and has even been proposed as a prognosticator of complicated pregnancies [46].

While the changes in the VWFpp:Ag and FVIII:VWF ratios observed during pregnancy are consistent with acquired qualitative changes in VWF, more direct evidence of acquired VWF structural changes are apparent in the analysis of VWF multimers (Figs 3 and 4). In most of the pregnancies analyzed, VWF multimer patterns changed with a loss of higher order multimers and altered VWF triplet structure. These changes in VWF multimers suggest acquired differences in VWF function [6], [47]. One mechanism by which VWF multimer patterns could change would be by altered proteolysis, despite no change in ADAMTS13 activity by our assay. The VWF itself could have acquired an increased propensity to cleavage by ADAMTS13 by virtue of acquired molecular changes during pregnancy, or the differences in multimer structure may reflect altered proteolysis by other plasma proteases. Another possibility is that a prolongation in survival of VWF provides additional opportunity for VWF to be cleaved by ADAMTS13, thereby reducing multimer size and shifting triplet structure [48]. Another clue that distinct molecular changes in VWF can be acquired during pregnancy lies in the shift of the isoelectric focus point. To our knowledge, this shift has not been detected before and the molecular nature of this distinct change is as yet unknown. We speculate that this shift in VWF IEF reflects altered posttranslational modifications, most likely carbohydrate alterations, but other molecular changes can influence IEF patterns [49].

We conclude that VWF can progressively and reversibly acquire both quantitative and qualitative changes over the course of pregnancy. We propose a scenario by which pregnancy results in increased VWF production, prolonged VWF survival, and acquired VWF structural changes. Such changes could be adaptive in anticipation of the acute hemostatic challenges posed by delivery by increasing the overall quantity of VWF, yet attenuate the risk of thrombosis during pregnancy through the loss of higher order VWF multimers [50]. Furthermore, the more modest elevations in FVIII observed relative to VWF may somewhat diminish the risk of spontaneous thrombosis or vascular pathology which can accompany high FVIII levels in other settings [51], [52].

## Supporting Information

Table S1 **Individual subject data for VWF-associated parameters measured during pregnancy and at baseline.**
(DOCX)
