# Supplementary material for: Complex Changes in von Willebrand Factor-Associated Parameters Are Acquired during Uncomplicated Pregnancy
Source: PLoS One. 2014 Nov 19;9(11):e112935. doi: 10.1371/journal.pone.0112935 (PMC4237360; doi:10.1371/journal.pone.0112935)
Supplement: Table S1 — Individual subject data for VWF-associated parameters measured during pregnancy and at baseline. (DOCX) [file pone.0112935.s001.docx]

**Table S1. Individual subject data for VWF-associated parameters measured during pregnancy and at baseline.**

| **Sample ID** | **Predicted ABO Type^1^** | **Δ HMW Multimer^2^** | **Δ Triplet Multimer^2^** | **Draw** | **VWF:Ag** | **VWFpp** | **FVIII** | **ADAMTS13 Activity** | **VWFpp:Ag Ratio** | **FVIII:VWF Ratio** |
| --- | --- | --- | --- | --- | --- | --- | --- | --- | --- | --- |
| NOP 090A | O | Y | Y | 2nd | 75 | 113 | 111 | -- | 1.5 | 0.7 |
|  |  |  |  | 3rd | 154 | 99 | 176 | 153 | 0.6 | 0.9 |
|  |  |  |  | POST | 60 | 104 | 90 | 150 | 1.8 | 0.7 |
| NOP 104A | A | N | N | 1st | 151 | 179 | 168 | -- | 1.2 | 0.9 |
|  |  |  |  | 3rd | 254 | 217 | 209 | 111 | 0.9 | 1.2 |
|  |  |  |  | POST | 109 | 171 | 105 | 147 | 1.6 | 1.0 |
| NOP 106A | O | N | Y | PRE | 67 | 124 | 96 | -- | 1.8 | 0.7 |
|  |  |  |  | 1st | 60 | 88 | 115 | -- | 1.5 | 0.5 |
|  |  |  |  | 2nd | 97 | 120 | 121 | -- | 1.2 | 0.8 |
|  |  |  |  | 3rd | 333 | 185 | 132 | 121 | 0.6 | 2.5 |
|  |  |  |  | POST | 105 | 115 | 89 | 116 | 1.1 | 1.2 |
| NOP 135A | O | Y | Y | 3rd | 259 | 148 | 187 | 136 | 0.6 | 1.4 |
|  |  |  |  | POST | 80 | 81 | 76 | 122 | 1.0 | 1.1 |
| NOP 143A | B | N | -- | PRE | 105 | 155 | 158 | -- | 1.5 | 0.7 |
|  |  |  |  | 1st | 169 | 132 | 170 | -- | 0.8 | 1.0 |
|  |  |  |  | 3rd | 259 | 188 | 228 | 113 | 0.7 | 1.1 |
|  |  |  |  | POST | 125 | 183 | 145 | 112 | 1.5 | 0.9 |
| NOP 146A | O | Y | Y | PRE | 95 | 172 | 77 | -- | 1.8 | 1.2 |
|  |  |  |  | 1st | 99 | 101 | 83 | -- | 1.0 | 1.2 |
|  |  |  |  | 2nd | 105 | 139 | 131 | -- | 1.3 | 0.8 |
|  |  |  |  | 3rd | 94 | 109 | 105 | 102 | 1.2 | 0.9 |
|  |  |  |  | POST | 58 | 138 | 83 | 130 | 2.4 | 0.7 |
| NOP 153A | A | -- | -- | 1st | 151 | 152 | 196 | -- | 1.0 | 0.8 |
|  |  |  |  | 2nd | 213 | 140 | 190 | -- | 0.7 | 1.1 |
|  |  |  |  | 3rd | 212 | 182 | 228 | 115 | 0.9 | 0.9 |
|  |  |  |  | POST | 46 | 86 | 64 | 176 | 1.9 | 0.7 |
| NOP 155A | O | Y | Y | 1st | 80 | 148 | 95 | -- | 1.8 | 0.8 |
|  |  |  |  | 2nd | 104 | 112 | 159 | -- | 1.1 | 0.7 |
|  |  |  |  | 3rd | 219 | 185 | 198 | 99 | 0.8 | 1.1 |
|  |  |  |  | POST | 53 | 80 | 91 | 131 | 1.5 | 0.6 |
| NOP 165A | O | N | Y | 1st | 103 | 115 | 134 | -- | 1.1 | 0.8 |
|  |  |  |  | 2nd | 149 | 140 | 203 | -- | 0.9 | 0.7 |
|  |  |  |  | 3rd | 217 | 175 | 241 | 124 | 0.8 | 0.9 |
|  |  |  |  | POST | 83 | 97 | 141 | 157 | 1.2 | 0.6 |
| NOP 168A | O | Y | N | 2nd | 85 | 126 | 124 | -- | 1.5 | 0.7 |
|  |  |  |  | 3rd | 68 | 95 | 142 | 114 | 1.4 | 0.5 |
|  |  |  |  | POST | 74 | 146 | 102 | 114 | 2.0 | 0.7 |
| NOP 170A | B | -- | -- | 1st | 46 | 58 | 79 | -- | 1.3 | 0.6 |
|  |  |  |  | 3rd | 75 | 106 | 129 | 108 | 1.4 | 0.6 |
|  |  |  |  | POST | 45 | 110 | 117 | 115 | 2.4 | 0.4 |
| NOP 175A | O | Y | Y | 2nd | 122 | 134 | 147 | -- | 1.1 | 0.8 |
|  |  |  |  | 3rd | 261 | 215 | 336 | 160 | 0.8 | 0.8 |
|  |  |  |  | POST | 44 | 97 | 93 | 160 | 2.2 | 0.5 |
| NOP 190A | AB | Y | -- | PRE | 126 | 131 | 141 | -- | 1.0 | 0.9 |
|  |  |  |  | 1st | 212 | 221 | 248 | -- | 1.0 | 0.9 |
|  |  |  |  | 2nd | 235 | 254 | 312 | -- | 1.1 | 0.8 |
|  |  |  |  | 3rd | 337 | 281 | 295 | 122 | 0.8 | 1.1 |
|  |  |  |  | POST | 167 | 200 | 178 | 114 | 1.2 | 0.9 |
| NOP 208A | B | N | N | PRE | 102 | 149 | 98 | -- | 1.5 | 1.0 |
|  |  |  |  | 1st | 138 | 209 | 122 | -- | 1.5 | 1.1 |
|  |  |  |  | 2nd | 103 | 126 | 105 | -- | 1.2 | 1.0 |
|  |  |  |  | 3rd | 153 | 185 | 152 | 131 | 1.2 | 1.0 |
|  |  |  |  | POST | 94 | 181 | 92 | 117 | 1.9 | 1.0 |
| NOP 210A | A | Y | N | 2nd | 165 | 161 | 236 | -- | 1.0 | 0.7 |
|  |  |  |  | 3rd | 197 | 173 | 259 | 93 | 0.9 | 0.8 |
|  |  |  |  | POST | 113 | 132 | 160 | 113 | 1.2 | 0.7 |
| NOP 213A | AB | -- | -- | 1st | 198 | 192 | 147 | -- | 1.0 | 1.4 |
|  |  |  |  | 2nd | 201 | 196 | 216 | -- | 1.0 | 0.9 |
|  |  |  |  | 3rd | 292 | 181 | 243 | 101 | 0.6 | 1.2 |
|  |  |  |  | POST | 131 | 166 | 146 | 111 | 1.3 | 0.9 |
| NOP 221A | A | -- | -- | 1st | 101 | 135 | 107 | -- | 1.3 | 0.9 |
|  |  |  |  | 2nd | 90 | 104 | 114 | -- | 1.2 | 0.8 |
|  |  |  |  | 3rd | 178 | 158 | 148 | 98 | 0.9 | 1.2 |
|  |  |  |  | POST | 60 | 130 | 86 | 121 | 2.2 | 0.7 |
| NOP 222A | A | -- | -- | 3rd | 193 | 191 | 225 | 113 | 1.0 | 0.9 |
|  |  |  |  | POST | 67 | 105 | 129 | 126 | 1.6 | 0.5 |
| NOP 237A | A | N | N | PRE | 95 | 123 | 140 | -- | 1.3 | 0.7 |
|  |  |  |  | 1st | 103 | 165 | 164 | -- | 1.6 | 0.6 |
|  |  |  |  | 2nd | 135 | 176 | 249 | -- | 1.3 | 0.5 |
|  |  |  |  | 3rd | 181 | 135 | 231 | 162 | 0.7 | 0.8 |
|  |  |  |  | 38wk | 128 | 115 | 187 | -- | 0.9 | 0.7 |
|  |  |  |  | POST | 71 | 159 | 151 | 173 | 2.2 | 0.5 |
| NOP 242A | B | -- | Y | 2nd | 101 | 105 | 164 | -- | 1.0 | 0.6 |
|  |  |  |  | 3rd | 238 | 192 | 195 | 149 | 0.8 | 1.2 |
|  |  |  |  | POST | 69 | 104 | 222 | 134 | 1.5 | 0.3 |
| NOP 244A | B | Y | N | 2nd | 182 | 178 | 224 | -- | 1.0 | 0.8 |
|  |  |  |  | 3rd | 193 | 153 | 220 | 131 | 0.8 | 0.9 |
|  |  |  |  | POST | 82 | 124 | 131 | 90 | 1.5 | 0.6 |
| NOP 245A | B | Y | Y | 1st | 110 | 117 | 148 | -- | 1.1 | 0.7 |
|  |  |  |  | 2nd | 157 | 117 | 205 | -- | 0.7 | 0.8 |
|  |  |  |  | 3rd | 173 | 160 | 237 | 119 | 0.9 | 0.7 |
|  |  |  |  | POST | 84 | 136 | 174 | 110 | 1.6 | 0.5 |
| NOP 248A | A | Y | Y | 2nd | 181 | 156 | 180 | -- | 0.9 | 1.0 |
|  |  |  |  | 3rd | 177 | 177 | 188 | 189 | 1.0 | 0.9 |
|  |  |  |  | POST | 55 | 154 | 105 | 247 | 2.8 | 0.5 |
| NOP 249A | A | -- | N | PRE | 100 | 167 | 141 | -- | 1.7 | 0.7 |
|  |  |  |  | 1st | 127 | 127 | 141 | -- | 1.0 | 0.9 |
|  |  |  |  | 2nd | 83 | 93 | 119 | -- | 1.1 | 0.7 |
|  |  |  |  | 3rd | 243 | 168 | 195 | 193 | 0.7 | 1.2 |
|  |  |  |  | POST | 91 | 208 | 123 | 190 | 2.3 | 0.7 |
| NOP 250A | A | Y | Y | 2nd | 123 | 106 | 134 | -- | 0.9 | 0.9 |
|  |  |  |  | 3rd | 121 | 72 | 124 | 157 | 0.6 | 1.0 |
|  |  |  |  | POST | 94 | 97 | 112 | 83 | 1.0 | 0.8 |
| NOP 253A | O | -- | -- | PRE | 64 | 109 | 93 | -- | 1.7 | 0.7 |
|  |  |  |  | 2nd | 117 | 154 | 151 | -- | 1.3 | 0.8 |
|  |  |  |  | 3rd | 131 | 99 | 158 | -- | 0.8 | 0.8 |
| NOP 261A | A | Y | Y | 1st | 118 | 128 | 119 | -- | 1.1 | 1.0 |
|  |  |  |  | 2nd | 88 | 111 | 125 | -- | 1.3 | 0.7 |
|  |  |  |  | 3rd | 125 | 123 | 175 | 88 | 1.0 | 0.7 |
|  |  |  |  | POST | 43 | 135 | 104 | 114 | 3.1 | 0.4 |
| NOP 268A | B | ND | N | 3rd | 305 | 201 | 193 | 119 | 0.7 | 1.6 |
|  |  |  |  | POST | 118 | 176 | 130 | 98 | 1.5 | 0.9 |
| NOP 269A | B | -- | -- | PRE | 106 | 113 | 119 | -- | 1.1 | 0.9 |
|  |  |  |  | 1st | 96 | 129 | 140 | -- | 1.3 | 0.7 |
|  |  |  |  | 2nd | 172 | 136 | 174 | -- | 0.8 | 1.0 |
|  |  |  |  | 3rd | 207 | 108 | 215 | 104 | 0.5 | 1.0 |
|  |  |  |  | 38wk | 200 | 145 | 225 | -- | 0.7 | 0.9 |
|  |  |  |  | POST | 82 | 111 | 140 | 96 | 1.4 | 0.6 |
| NOP 270A | B | Y | Y | 2nd | 184 | 194 | 302 | -- | 1.1 | 0.6 |
|  |  |  |  | 3rd | 287 | 189 | 358 | 119 | 0.7 | 0.8 |
|  |  |  |  | 38wk | 373 | 258 | 291 | -- | 0.7 | 1.3 |
|  |  |  |  | POST | 94 | 141 | 164 | 90 | 1.5 | 0.6 |
| NOP 274A | A | Y | Y | PRE | 91 | 93 | 131 | -- | 1.0 | 0.7 |
|  |  |  |  | 2nd | 154 | 114 | 195 | -- | 0.7 | 0.8 |
|  |  |  |  | 3rd | 177 | 122 | 249 | 114 | 0.7 | 0.7 |
|  |  |  |  | POST* | 60 | 71 | 156 | 103 | 1.2 | 2.6 |
| NOP 277A | A | -- | -- | 1st | 163 | 185 | 120 | -- | 1.1 | 1.4 |
|  |  |  |  | 2nd | 145 | 135 | 122 | -- | 0.9 | 1.2 |
|  |  |  |  | 3rd | 167 | 178 | 190 | 110 | 1.1 | 0.9 |
|  |  |  |  | POST | 55 | 127 | 84 | 142 | 2.3 | 0.7 |
| NOP 282A | O | -- | -- | PRE | 77 | 98 | 70 | -- | 1.3 | 1.1 |
|  |  |  |  | 1st | 132 | 162 | 89 | -- | 1.2 | 1.5 |
|  |  |  |  | 2nd | 82 | 98 | 110 | -- | 1.2 | 0.7 |
|  |  |  |  | 3rd | 129 | 113 | 118 | -- | 0.9 | 1.1 |
| NOP 287A | A | -- | -- | 2nd | 168 | 153 | 188 | -- | 0.9 | 0.9 |
|  |  |  |  | 3rd | 135 | 136 | 163 | 137 | 1.0 | 0.8 |
|  |  |  |  | 38wk | 252 | 212 | 205 | -- | 0.8 | 1.2 |
|  |  |  |  | POST | 77 | 116 | 105 | 114 | 1.5 | 0.7 |
| NOP 292A | O | -- | -- | 2nd | 123 | 159 | 144 | -- | 1.3 | 0.9 |
|  |  |  |  | 3rd | 93 | 92 | 149 | 75 | 1.0 | 0.6 |
|  |  |  |  | 38wk | 328 | 248 | 252 | -- | 0.8 | 1.3 |
|  |  |  |  | POST | 46 | 125 | 87 | 88 | 2.7 | 0.5 |
| NOP 294A | A | -- | -- | 1st | 131 | 170 | 123 | -- | 1.3 | 1.1 |
|  |  |  |  | 2nd | 172 | 152 | 168 | -- | 0.9 | 1.0 |
|  |  |  |  | 3rd | 192 | 132 | 246 | 80 | 0.7 | 0.8 |
|  |  |  |  | POST | 90 | 195 | 134 | 89 | 2.2 | 0.7 |
| NOP 301A | O | -- | -- | PRE | 80 | 133 | 91 | -- | 1.7 | 0.9 |
|  |  |  |  | 1st | 66 | 109 | 77 | -- | 1.7 | 0.8 |
|  |  |  |  | 2nd | 82 | 122 | 135 | -- | 1.5 | 0.6 |
|  |  |  |  | 3rd | 109 | 118 | 228 | 89 | 1.1 | 0.5 |
|  |  |  |  | 38wk | 207 | 209 | 166 | -- | 1.0 | 1.2 |
|  |  |  |  | POST | 59 | 152 | 79 | 141 | 2.6 | 0.7 |
| NOP 303A | AB | -- | -- | PRE | 148 | 170 | 128 | -- | 1.1 | 1.2 |
|  |  |  |  | 1st | 154 | 168 | 171 | -- | 1.1 | 0.9 |
|  |  |  |  | 2nd | 251 | 187 | 233 | -- | 0.7 | 1.1 |
|  |  |  |  | 38wk | 279 | 192 | 209 | -- | 0.7 | 1.3 |
|  |  |  |  | POST | 115 | 137 | 145 | -- | 1.2 | 0.8 |
| NOP 304A | A | Y | Y | 1st | 85 | 136 | 109 | -- | 1.6 | 0.8 |
|  |  |  |  | 2nd | 99 | 129 | 124 | -- | 1.3 | 0.8 |
|  |  |  |  | 3rd | 221 | 218 | 176 | 78 | 1.0 | 1.3 |
|  |  |  |  | POST | 64 | 96 | 76 | 95 | 1.5 | 0.8 |
| NOP 316A | B | -- | -- | 38wk | 228 | 181 | 243 | -- | 0.8 | 0.9 |
|  |  |  |  | POST | 83 | 141 | 115 | -- | 1.7 | 0.7 |
| NOP 319A | A | -- | -- | 2nd | 128 | 148 | 132 | -- | 1.2 | 1.0 |
|  |  |  |  | 3rd | 65 | 113 | 92 | 100 | 1.7 | 0.7 |
|  |  |  |  | POST | 73 | 127 | 108 | 112 | 1.7 | 0.7 |
| NOP 322A | A | Y | Y | 1st | 92 | 117 | 135 | -- | 1.3 | 0.7 |
|  |  |  |  | 2nd | 196 | 153 | 249 | -- | 0.8 | 0.8 |
|  |  |  |  | 3rd | 263 | 197 | 270 | 91 | 0.7 | 1.0 |
|  |  |  |  | 38wk | 545 | 460 | 275 | -- | 0.8 | 2.0 |
|  |  |  |  | POST | 81 | 124 | 122 | 79 | 1.5 | 0.7 |
| NOP 324A | B | -- | -- | PRE | 95 | 99 | 111 | -- | 1.0 | 0.9 |
|  |  |  |  | 2nd | 211 | 139 | 141 | -- | 0.7 | 1.5 |
|  |  |  |  | 3rd | 183 | 138 | 137 | -- | 0.8 | 1.3 |
| NOP 327A | B | -- | -- | 2nd | 146 | 147 | 167 | -- | 1.0 | 0.9 |
|  |  |  |  | 3rd | 200 | 206 | 207 | 140 | 1.0 | 1.0 |
|  |  |  |  | POST | 100 | 127 | 121 | 126 | 1.3 | 0.8 |
| NOP 330A | B | -- | -- | 2nd | 95 | 137 | 139 | -- | 1.4 | 0.7 |
|  |  |  |  | 3rd | 87 | 128 | 166 | -- | 1.5 | 0.5 |
|  |  |  |  | 38wk | 126 | 135 | 159 | 67 | 1.1 | 0.8 |
|  |  |  |  | POST | 40 | 110 | 102 | 60 | 2.7 | 0.4 |
| NOP 334A | O | -- | -- | 3rd | 135 | 142 | 197 | 98 | 1.1 | 0.7 |
|  |  |  |  | 38wk | 158 | 131 | 200 | -- | 0.8 | 0.8 |
|  |  |  |  | POST | 95 | 178 | 162 | 85 | 1.9 | 0.6 |
| ^1^ABO blood type assigned by *ABO* SNP genotype; ^2^A shift (Δ) in VWF multimers (in patterns shown in Figs 3 and 4) between baseline and pregnancy is indicated with “Y” when there was consensus of two independent observers. “N” indicates one or both observers determined there to be no clear change in multimer patterns. “--“ Indicates missing data (not measured) | | | | | | | | | | |
